# Incidence of Symptoms and Accidents During Baths and Showers Among the Japanese General Public

**DOI:** 10.2188/jea.JE20100136

**Published:** 2011-07-05

**Authors:** Shinya Hayasaka, Yosuke Shibata, Tatsuya Noda, Yasuaki Goto, Toshiyuki Ojima

**Affiliations:** 1 Department of Community Health and Preventive Medicine, Hamamatsu University School of Medicine, Hamamatsu, Japan; 2 Japan Health and Research Institute, Tokyo, Japan

**Keywords:** baths, shower, incidence, accidents, population-based study

## Abstract

**Background:**

Bathing is a deeply ingrained custom among Japanese; however, data on the incidence rate of symptoms and accidents during bathing have not yet been reported for the Japanese general public.

**Methods:**

We conducted a population-based cross-sectional study of 617 Japanese adults who attended a specialized health checkup. Participants completed a self-administered questionnaire to assess weekly frequencies of bathtub bathing and showering and the frequency of symptoms/accidents (falling, loss of consciousness, and other) during these activities in the past year. We calculated the incidence rates of accidents per 10 000 baths/showers and 95% confidence intervals (CIs) and compared the clinical characteristics of participants who had symptoms/accidents with those who did not.

**Results:**

The incidence rates of accidents per 10 000 bathtub baths and showers were 0.43 (95% CI: 0.22–0.84) and 0.24 (95% CI: 0.04–1.37). Although these rates are low, there were 740 000 bathtub bathing-related accidents in Japan, due to the fact that bathing is an almost-daily habit. There was no significant difference in clinical characteristics between groups

**Conclusions:**

We collected basic information on the incidence of bathing-related accidents in Japan. Falls and loss of consciousness during bathing or showering can potentially lead to a serious accident, so the general public should be educated about the possibility of such accidents during bathing.

## INTRODUCTION

Bathing is a custom that is deeply ingrained in the life of Japanese people. Unlike their European and American counterparts,^1^ many Japanese are thought to bathe in a bathtub almost daily; however, there are few reports on the present bathing habits of the Japanese general public. Bathing-related symptoms and accidents are frequently reported, which has led allied medical associations to highlight the dangers associated with bathing and submit an official statement to the Japanese Ministry of Health, Labour and Welfare regarding the need for a systematic investigation of the causes of such accidents.^2^ Previous reports on bathing-related accidents include a study that used existing data on accidental bathtub drowning deaths and found that approximately 3500 people drown in household bathtubs every year,^3^ a medicolegal study of deaths during bathing,^4^ an investigation of accidents occurring at hot spring resorts,^5^ an investigation of cases involving emergency medical transport,^6^ and a report on accidents occurring in welfare service settings.^7^ The only available incidence rate for bathing-related accidents is one based on such welfare services, as reported by us previously.^8^ Basic data on the incidence rate among the general public have not been reported in Japan. The present study aimed to investigate bathing habits and the incidence rate of bathing-related symptoms/accidents among the Japanese general public.

## METHODS

This cross-sectional study enrolled adults aged 40 to 74 years who underwent a specialized health checkup provided in October 2008 in the district of Kawane in Shimada city, Shizuoka. The investigation utilized self-administered questionnaires, measurement of height and weight, blood pressure examination, and blood testing for triglyceride, low-density lipoprotein cholesterol, high-density lipoprotein cholesterol, L-aspartate aminotransferase, L-alanine aminotransferase, and hemoglobin A1c, based on a program standardized by the Japanese Ministry of Health, Labour and Welfare.^9^ From a total of 1319 eligible adults, 617 who actually completed the health checkup and gave consent participated in the study. Participants completed a self-administered questionnaire to assess the weekly frequencies of bathtub bathing and showering during summer (July through September) and winter (December through February). In addition, we investigated the frequency of symptoms/accidents (falling, loss of consciousness, pallor, nausea/vomiting, and other) during bathtub bathing and showering in the past year: the list of symptoms/accidents consisted of those frequently identified in previous research.^7^

First, we calculated the weekly frequencies of bathtub bathing and showering by averaging the frequencies between the 2 seasons, after which we assessed the incidence of each type of accident during bathtub bathing and showering in the past year for all participants and in 2 groups divided by the median age of the participants (ie, 66 years). Next, based on the weekly frequencies of bathtub bathing and showering, we estimated the overall frequencies of bathtub bathing and showering during the past year for all participants. We then divided the total number of accidents by the annual frequencies of bathtub bathing and showering for all participants and calculated the incidence rates and 95% confidence intervals (CIs) of accidents per 10 000 baths/showers. We calculated 95% CIs using a binomial distribution according to Wilson’s method.^10^ Then, to estimate the annual frequencies of bathtub bathing and showering among Japanese aged 40 to 74 years, we used the 2008 Vital Statistics of Japan to determine how many Japanese were included in this age group.^11^ By multiplying these estimates by the incidence rates for accidents (which were obtained earlier), we estimated the total annual numbers of accidents that were associated with bathtub bathing and showering in Japanese aged 40 to 74 years.

After dividing the participants into 2 groups according to their accident status, ie, cases with and without bath-related accidents, we used the *t*-test to compare mean values for age, body mass index, blood pressure, and blood chemistry findings and Fisher’s exact test to compare differences in sex, medication use, and past medical history.

The study protocol was approved by the institutional review board (IRB) of Hamamatsu University School of Medicine (No. 20-55). The nature of the study was explained to the participants using written statements approved by the IRB. Voluntary informed consent was obtained from all participants before beginning the study. Completion of the questionnaire was deemed as indicating consent to participate in the study. Statistical analyses were performed using the Statistical Package for Social Science (SPSS) for Windows (SPSS Japan Inc., version 17.0, Tokyo, Japan).

## RESULTS

All 617 individuals (266 men, 351 women) who had completed the health checkup and were recruited for the study agreed to participate. The frequencies of bathtub bathing and showering were calculated based on summer and winter frequencies (mean ± SD) of 5.82 ± 2.09 and 1.29 ± 1.81 times per week, respectively (Table 1). The overall annual numbers of bathtub baths and showers were estimated based on the weekly frequencies and were approximately 190 000 and 41 000, respectively. Of the 617 individuals, 588 completed a self-administered questionnaire about accidents. Of these, 5 reported a total of 8 bathtub bathing-related accidents over the past year, including 5 cases of facial pallor, 2 cases of loss of consciousness, and 1 case of falling. In contrast, there was only 1 shower-related accident (loss of consciousness). The incidence rates of accidents per 10 000 bathtub baths and showers were 0.43 (95% CI: 0.22–0.84) and 0.24 (95% CI: 0.04–1.37), respectively. According to estimates based on the Japanese population of adults aged 40 to 74 years, people in this age group bathe a total of 1.7 × 10^10^ times per year and have a total of 740 000 (95% CI: 380 000 to 1 500 000) accidents. They shower 3.8 × 10^9^ times per year and have a total of 93 000 (95% CI: 16 000 to 530 000) accidents.

**Table 1.** Frequency and incidence of accidents during bathtub bathing and showering

	Bathtub bathing	Showering
Weekly frequency (mean ± SD, *n* = 617)	5.82 ± 2.09	1.29 ± 1.81
age <67 y (*n* = 329)	5.80 ± 2.12	1.49 ± 1.89
age ≥67 y (*n* = 288)	5.86 ± 2.05	1.06 ± 1.69
Overall annual frequency (estimate)	1.9 × 10^5^	4.1 × 10^4^
age <67 y	9.9 × 10^4^	2.5 × 10^4^
age ≥67 y	8.8 × 10^4^	1.6 × 10^4^
Number of reported symptoms/accidents in the past year (*n* = 588)		
Any symptom/accident	8	1
Any symptom/accident (age <67 y)	6	1
Any symptom/accident (age ≥67 y)	2	0
Pallor	5	0
Loss of consciousness	2	1
Falling	1	0
Nausea/Vomiting	0	0
Other	0	0
Incidence rate of symptoms/accidents per 10 000 baths/showers (95% CI)		
Any symptom/accident	0.43 (0.22–0.84)	0.24 (0.04–1.37)
Any symptom/accident (age <67 y)	0.60 (0.28–1.32)	0.39 (0.07–2.22)
Any symptom/accident (age ≥67 y)	0.23 (0.06–0.83)	0 (0–2.41)
Pallor	0.27 (0.11–0.63)	0 (0–0.93)
Loss of consciousness	0.11 (0.03–0.39)	0.24 (0.04–1.37)
Falling	0.05 (0.01–0.31)	0 (0–0.93)
Nausea/Vomiting	0 (0–0.21)	0 (0–0.93)
Other	0 (0–0.21)	0 (0–0.93)
Annual frequency in Japanese aged 40–74 years (estimate)	1.7 × 10^10^	3.8 × 10^9^
Total annual number of accidents across the Japanese population aged 40–74 years (estimate)	7.4 × 10^5^ (3.8 × 10^5^–1.5 × 10^6^)	9.3 × 10^4^ (1.6 × 10^4^–5.3 × 10^5^)

SD: standard deviation; CI: confidence interval.

A comparison of the characteristics of participants who did and did not have bath-related accidents are shown in Table 2. There was no significant difference in the characteristics of the 5 participants who had accidents and those who did not.

**Table 2.** Comparison of characteristics of participants who did and did not have bath-related accidents

	Accident (*n* = 5)	No accident (*n* = 583)	Total (*n* = 588)
Sex, *n* (%)			
Male	3 (60%)	250 (43%)	253 (43%)
Female	2 (40%)	333 (57%)	335 (57%)
Total	5 (100%)	583 (100%)	588 (100%)

Medication, *n* (%)			
Hypertension(+)	1 (20%)	217 (37%)	218 (37%)
Diabetes(+)	1 (20%)	37 (6%)	38 (7%)
Hyperlipidemia(+)	0 (0%)	130 (22%)	130 (22%)

Past history, *n* (%)			
Stroke(+)	0 (0%)	33 (6%)	33 (6%)
Ischemic heart disease(+)	0 (0%)	24 (4%)	24 (4%)
Chronic renal failure(+)	0 (0%)	0 (0%)	0 (0%)

Age, mean (SD), year	62.4 (10.1)	64.2 (8.0)	64.2 (8.0)
BMI, mean (SD), kg/m^2^	21.9 (1.7)	22.3 (3.5)	22.3 (3.5)
SBP, mean (SD), mm Hg	124.8 (22.4)	126.6 (20.0)	126.5 (20.0)
Triglyceride, mean (SD), mg/dl	86.6 (42.1)	100.7 (52.7)	100.6 (52.6)
LDL, mean (SD), mg/dl	135.2 (31.1)	129.8 (30.6)	129.8 (30.6)
HDL, mean (SD), mg/dl	68.6 (23.0)	68.5 (16.2)	68.5 (16.2)
AST, mean (SD), U/l	35.2 (32.6)	24.9 (10.8)	25.0 (11.1)
ALT, mean (SD), U/l	52.8 (84.0)	21.1 (13.2)	21.4 (15.1)
Hemoglobin A1c, %	6.0 (1.6)	5.4 (0.6)	5.4 (0.7)

BMI: Body mass index; SBP: Systolic blood pressure; LDL: low-density lipoprotein cholesterol; HDL: high-density lipoprotein cholesterol; AST: L-aspartate aminotransferase; ALT: L-alanine aminotransferase.

## DISCUSSION

The present study is the first to ascertain the incidence rate of bathing-related accidents among the Japanese general public. The incidence rate in the present study was somewhat higher than that noted in an investigation of public welfare facilities for the aged (0.067 and 0.204 per 10 000 in-facility baths and home-visit baths, respectively).^8^ The somewhat lower incidence rate of accidents in bathing services provided by welfare facilities may be attributable to the following facts. First, in welfare services, a physical checkup is routinely performed before bathing, which may prevent accidents. Second, bathing in the presence of a caregiver may allow for early detection of potential accidents and serious symptoms. Third, the incidence rate was likely to have been underreported in the previous study,^8^ as it was based only on the objective findings of caregivers.

In the present study, the incidence rate of accidents during showering was lower than that during bathtub bathing. It has been suggested that, as compared with bathtub bathing, the lower water pressure used during showering imposes a lower burden on the body.^12^ However, as habitual bathing is reported to be associated with good self-rated health and sleep quality,^13^ it would not be wise to suggest that bathtub bathing is, in general, more hazardous to general health than showering.

While the incidence rate of accidents in the present study is small, the total number of bathtub bathing-related accidents in Japan was as high as 740 000, because bathing is an almost-daily habit. Although no accidents resulted in death in this study, falls and loss of consciousness could potentially lead to a serious accident.^14^ One participant who reported falling in the bath lost consciousness twice. Therefore, the general public must be educated about accidents such as falls during bathing.

This study has several limitations. First, the use of self-report questionnaires may have caused underreporting due to obscured memory. Second, the sample size was relatively small, and the study was conducted within 1 geographic region. It is very difficult to use a small sample in a specific area to estimate the incidence for the general population. To determine the characteristics of the accident group, we analyzed the relationship between disease history and the results of the health checkup using additional data from the health checkup. However, there were no characteristic findings. Third, participants had all sought a health checkup, suggesting possible selection bias. A previous study reported high incidence rates of drowning among the very old.^3^ It is possible that, in the present study, recall bias explains why incidence rates were lower among the very old than among middle-aged adults. Although there were some limitations in the present report, it is the first to collect information on the incidence of bathing-related accidents among the Japanese general public.
